# Research on Frequency Doubling Effect of Thermoacoustic Speaker Based on Graphene Film

**DOI:** 10.3390/s21186030

**Published:** 2021-09-09

**Authors:** Licheng Deng, Xingyue He, Surong He, Qingying Ren, Jiang Zhao, Debo Wang

**Affiliations:** College of Electronic and Optical Engineering & College of Microelectronics, Nanjing University of Posts and Telecommunications, Nanjing 210023, China; dlc@njupt.edu.cn (L.D.); 1218023035@njupt.edu.cn (X.H.); 1219023540@njupt.edu.cn (S.H.); rqy@njupt.edu.cn (Q.R.); jzhao@njupt.edu.cn (J.Z.)

**Keywords:** frequency doubling effect, graphene film, thermoacoustic effect, thermoacoustic speaker, laser scribing technology

## Abstract

In this work, the frequency doubling effect of thermoacoustic speakers is studied, and a method is analyzed to suppress the frequency doubling effect. Three cases were analyzed by superimposing the DC bias on the AC excitation: (1) DC is less than AC; (2) DC is equal to AC; (3) DC is greater than AC. We found that the frequency doubling effect can be well suppressed by superimposing a larger DC excitation on the AC excitation. The laser scribing technology was used to prepare graphene film in only one step, and the screen printing technology was used to prepare conductive electrodes. The microphone and B&K system was used to record the sound pressure level and study the suppression of frequency doubling effect. Finally, the sound pressure levels with the three different kinds of excitations were measured. The measured results show that they have a good agreement with the theoretical results. The suppression effect will be better when DC amplitude is greater than AC amplitude. Therefore, this work has certain reference significance for the further study and application of thermoacoustic speakers.

## 1. Introduction

Nowadays, thermoacoustic speakers have been widely used in the field of sound [1,2,3]. The main reason is that the speaker based on the thermoacoustic effect does not have resonance phenomenon, and the frequency domain is wider than traditional speakers [4]. Three conditions have been put forward for thermoacoustic speakers to produce high SPL (sound pressure level) [5]:

(1) The conductive film is as thin as possible;
(2) The HCPUA (heat capacity per unit area) of the conductive film is as small as possible;
(3) The thermal conductivity of the conductive film is as large as possible.

With the development of nanomaterials and the nanomanufacturing industry, the thermoacoustic effect has been more widely studied. In 2014, Tian et al. used the laser scribing technology to show graphene earphones packaged in commercial earphones, with sounds ranging from 100 Hz to 50 kHz [6]. In 2015, Fei et al. prepared a graphene-foam-based speaker to study the effect of natural drying and freeze drying on generating sound [7]. In 2017, Zhang et al. used CVD (chemical vapor deposition) technology to prepare a kind of woven fabric composed of microribbons [8]. It can effectively produce sound under the sound-frequency electric field due to the thermoacoustic effect. In 2017, Tao et al. prepared a graphene-based intelligent artificial throat by a one-step laser induced method, realizing the functional integration of generating and detecting sound in a single device [9]. In 2018, Sbrockey et al. used several layers of graphene film to prepare thermoacoustic devices on a flexible plastic substrate, which can accurately reproduce sound including voice and music [10]. In 2019, Zhou et al. established a design criterion for the carbon nanotube film to improve the sound pressure level by minimizing the parallel layer spacing and filling up the multilayer CNT thin film with inert gas [11]. In 2020, Ahn et al. extracted carbon nanotube sheets from a vertically grown carbon nanotube forest, and found that the electrical performance of carbon nanotube sheet speakers and the heat spreading into the surrounding medium are closely related to the sound pressure levels [12]. Among them, graphene-based materials stand out for their thin thickness, low HCPUA and large thermal conductivity, becoming the preferred conductive thin film material for thermoacoustic speakers [13,14,15].

However, there is a frequency doubling effect of frequency distortion in the thermoacoustic effect, which means that the frequency of the generated sound is twice that of the input AC (alternating current). In the field of thermoacoustics, most research tends to focus on the improvement of sound pressure levels and electroacoustic conversion efficiency, but ignore the frequency doubling effect of thermoacoustic speakers [16,17,18,19,20,21]. Barnard et al. only briefly summarized the advantages and disadvantages of one method (superimposing the DC bias on the AC excitation) to suppress the frequency doubling effect. It did not conduct in-depth research on this method [22]. In our previous work, a theoretical model based on thermally induced energy density fluctuations was proposed, and the electrical-thermal-acoustic conversion behavior of graphene speakers was studied [23].

In this work, the frequency doubling effect of thermoacoustic speakers is studied, and a method is analyzed to suppress the frequency-doubling effect. LSG (laser-scribed graphene) was chosen to prepare the porous graphene-based thermoacoustic speaker in only one step. The method of superimposing DC (direct current) bias on AC was used to study the suppression of frequency doubling effect. In Section 2, the principle of frequency distortion caused by AC excitation and the suppression of frequency doubling effect by DC bias are studied. The following suppression conditions under different DC amplitudes are analyzed: (1) less than the AC amplitude; (2) equal to the AC amplitude; (3) greater than the AC amplitude. In Section 3, the fabrication process of the thermoacoustic speaker based on graphene film is given. Then, the sound pressure level generated by graphene speakers with different DC excitation superimposed on AC excitation is measured. Finally, this work is summarized in Section 4.

## 2. Theory and Model

**Figure 1:**
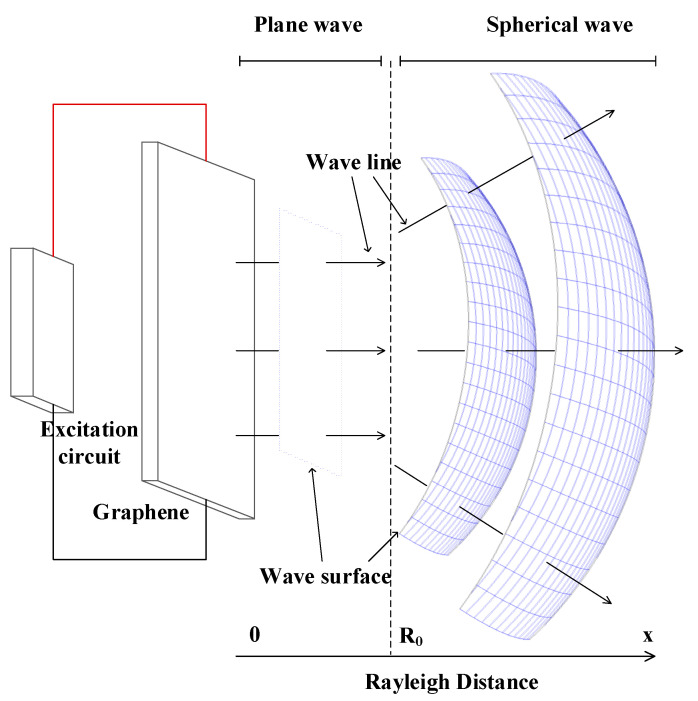
The working principle of thermoacoustic speaker.

The working principle of graphene thermoacoustic speakers is shown in Figure 1. When an alternating current is applied at both ends of graphene film, the surface of graphene transfers heat to the surrounding medium periodically, which causes the medium to expand and contract periodically. In the process, the electric energy is converted into thermal energy, and a periodic thermal gradient is formed between the thermoacoustic speaker and the surrounding air. The sound wave is produced by the periodic contraction and expansion of the medium caused by the thermal gradient. The sound wave is a plane wave in the Rayleigh distance (R_0_ = s/λ), and is a spherical wave outside the Rayleigh distance.

There is a frequency doubling effect in the process of electrical-thermal-acoustic conversion, which is due to the same heating effect of the positive and negative half period of AC excitation on the surrounding medium. The SPL generated by thermoacoustic effect is determined by the thermal energy, that is, by the electric power. If *AC* excitation is expressed as

(1) AC=Usinωt

where U is the amplitude of AC excitation, and ω is the frequency of *AC* excitation.

Then the electric power can be expressed as

(2) $$P_{A}=\frac{U^{2}}{R}\sin^{2}\omega t=\frac{U^{2}}{2R}(1-\cos2\omega t)$$

**Figure 2:**
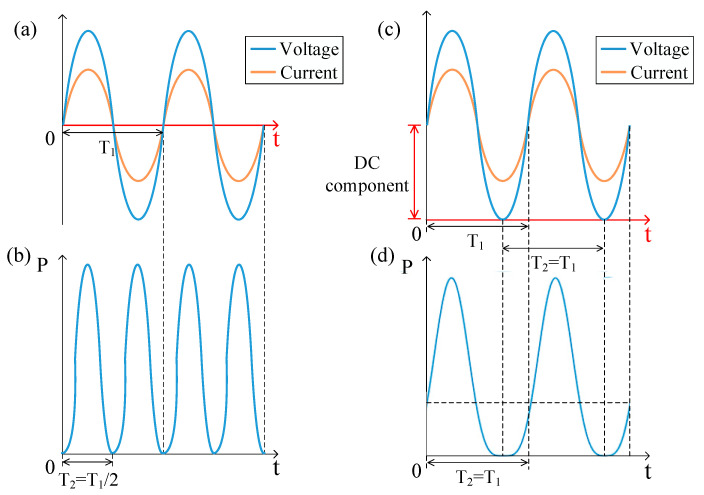
The curves of (**a**) voltage, current and (**b**) electric power under pure AC excitation, and the curves of (**c**) voltage, current and (**d**) electric power under AC and DC excitation.

It can be seen from Equation (2) that the frequency of electric power *P_A_* is twice that of *AC* excitation. In addition, the frequency of sound pressure is the same as that of electric power, so the frequency of sound pressure is twice that of *AC* excitation, as shown in Figure 2a,b. In order to suppress the frequency doubling effect, a *DC* bias excitation is used to superimpose the *AC* excitation.

The AC excitation and the DC bias excitation are respectively expressed as

(3) $$AC=U_{1}\sin\omega t$$

(4) $$DC=U_{2}$$

The electric power is expressed as

(5) $$\begin{array}{cc} P_{\left(A+D\right)} & =\frac{{\left(U_{1}\sin\omega t+U_{2}\right)}^{2}}{R}\\ & =\frac{U_{1}{}^{2}}{2R}(1-\cos2\omega t)+\frac{2U_{1}U_{2}}{R}\sin\omega t+\frac{U_{2}{}^{2}}{R} \end{array}$$

According to Equation (5), the single frequency component of electric power is

(6) $$P_{1}=\frac{U_{2}{}^{2}}{R}+\frac{2U_{1}U_{2}}{R}\sin\omega t$$

And the double frequency component of electric power is

(7) $$P_{2}=\frac{U_{1}{}^{2}}{2R}(1-\cos2\omega t)$$

The SPL is determined by the total power P_(A+D)_ = P1+P2. It can be predicted that the frequency of SPL is mainly determined by the relative magnitude of the single frequency component and the double frequency component of total electric power. The principle of suppressing the frequency doubling effect is shown in Figure 2c,d. The frequency doubling effect can be neglected when a large DC is applied.

In order to study the method to suppress the frequency doubling effect, we investigated the relationship between DC and AC. Keeping AC = 5 V, the DC amplitude was studied in three cases: (1) less than the AC amplitude; (2) equal to the AC amplitude; (3) greater than the AC amplitude.

**Figure 3:**
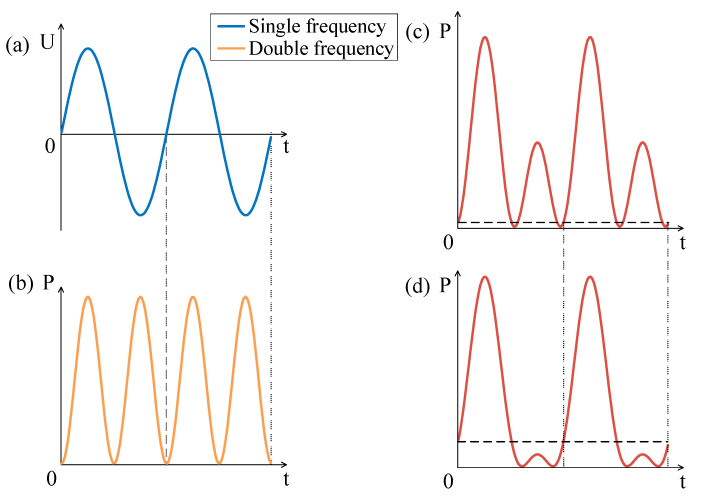
(**a**) AC excitation with amplitude of 5 V. Electric power with (**b**) AC = 5 V; (**c**) AC = 5 V and DC = 1 V; (**d**) AC = 5 V and DC = 3 V.

Firstly, we investigated the case that the amplitude of DC is less than that of AC. The AC excitation is shown in Figure 3a, that is, the single frequency curve. The electric power under pure AC excitation is shown in Figure 3b, that is, the double frequency curve. With the increasing of DC bias (as shown in Figure 3c DC = 1 V and Figure 3d DC = 3 V), it is obvious that the frequency doubling effect is gradually suppressed. However, as long as the DC amplitude is less than the AC amplitude, the frequency doubling effect will still exist.

**Figure 4:**
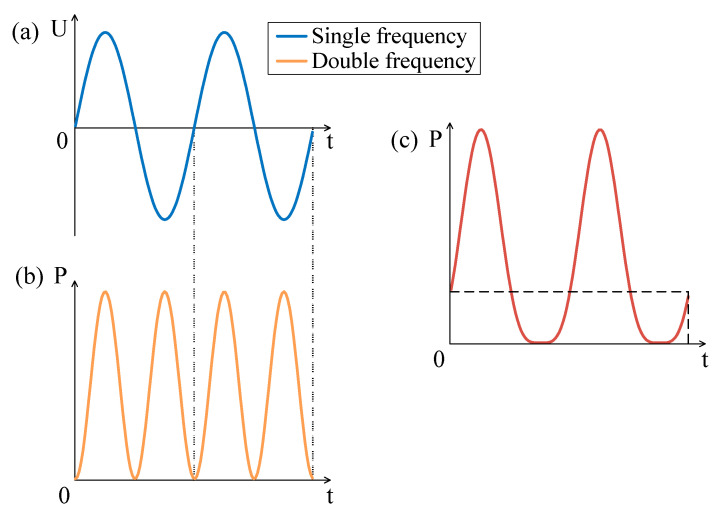
(**a**) AC excitation with amplitude of 5 V. Electric power with (**b**) AC = 5 V; (**c**) AC = 5 V and DC = 5 V.

Then, we studied the case of the amplitude of DC being equal to that of AC. It can be seen from Figure 4c that the frequency doubling effect is suppressed and the curve of electric power is more similar to that of the single frequency, but there is still distortion.

**Figure 5:**
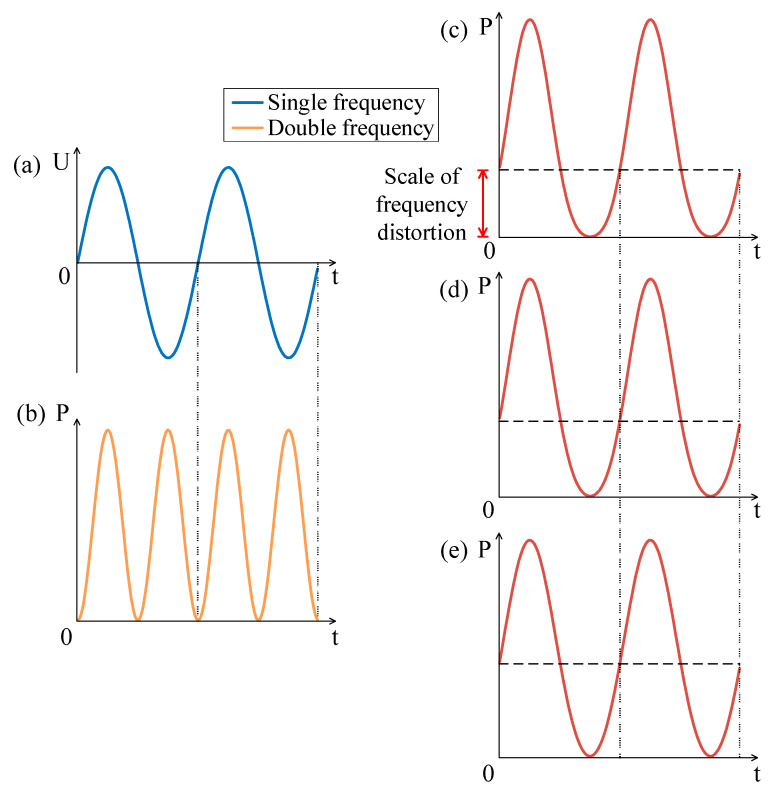
(**a**) AC excitation with amplitude of 5 V. Electric power with (**b**) AC = 5 V; (**c**) AC = 5 V and DC = 7 V; (**d**) AC = 5 V and DC = 9 V; (**e**) AC = 5 V and DC = 20 V.

Finally, we studied the frequency doubling effect when the amplitude of DC is larger than the amplitude of AC. It can be seen that with the increasing of DC amplitude, the distortion of the curve becomes smaller (as shown in Figure 5c DC = 7 V, Figure 5d DC = 9 V and Figure 5e DC = 20 V). The scale of distortion is defined in Figure 5c. The closer the scale is to half of the amplitude, the smaller the distortion is. As shown in Figure 5c,d, it can be found that when DC excitation is slightly larger than AC excitation, there will still be some distortion. Only when DC excitation is several times that of AC excitation, the frequency doubling effect is completely suppressed as shown Figure 5e. Generally, when the DC amplitude is larger than the AC amplitude, the frequency doubling effect can be ignored.

## 3. Design and Fabrication

**Figure 6:**
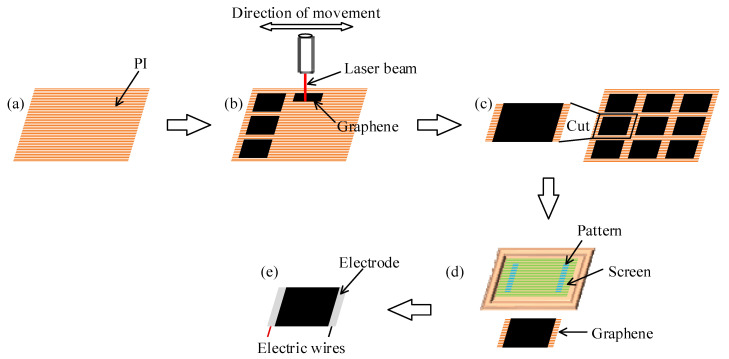
Fabrication process of the thermoacoustic speaker. (**a**) PI substrate; (**b**) Laser printing; (**c**) Cut the PI film; (**d**) Screen printing conductive silver glue; (**e**) Adding wires.

Laser scribing technology was chosen to prepare graphene thermoacoustic speakers in only one step [24]. The technology can be carried out directly in the air environment without high temperature or preliminary preparation [25,26]. The designed graphene array diagram was firstly imported into the Scarve software. As shown in Figure 6a, after the PI (polyimide) film was prepared, the laser beam was adjusted and focused on the PI film, and the scribing process was automatically completed as shown in Figure 6b. Kapton PI films were produced by the DuPont Company. The thickness of the PI films was 75 μm. The power of the laser was 10 W. The CO_2_ concentration of the CO_2_ laser was 20%, and N_2_ concentration of the CO_2_ laser was 15%. Then the desired shape or size of graphene was cut from the PI film as shown in Figure 6c. A certain width of PI film on both sides of the graphene was reserved, which was convenient for the preparation of conductive electrodes.

Screen printing was used to prepare conductive electrodes. The graphene film was fixed and aligned with the pattern on the screen. A squeegee with conductive silver glue was used to apply a certain pressure on the pattern, and the squeegee was moved evenly toward the bottom of the screen, as shown in Figure 6d, so that the pattern was completely printed on the surface of graphene film. The conductive electrodes’ pattern prepared by screen printing was complete, uniform and appropriate in size, which eliminated the problem of a rough surface and uneven distribution, and reduces the electric power loss caused by rough electrodes. As shown in Figure 6e, the thermoacoustic speaker was made by adding wires to the electrodes at both ends of the graphene film.

## 4. Measurements and Discussions

**Figure 7:**
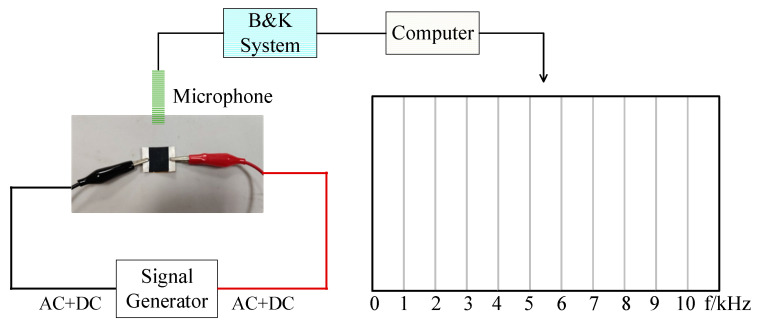
The diagram of measurement platform.

The measurement of the thermoacoustic speaker was undertaken in the anechoic chamber. The measurement platform is shown in Figure 7. A signal generator was used to provide excitation AC or (AC + DC) for the thermoacoustic speaker, and the B&K system connected to a microphone was used to collect and analyze the acoustic signal. The computer connected to the B&K system was used to display the parameters of the collected sound signal, such as SPL and frequency.

For pure AC excitation, for example, the frequency of the signal generator was adjusted to 1 kHz, and the computer only displayed the output sound pressure of 2 kHz. For AC/DC superimposed excitation, the frequency of the signal generator was also adjusted to 1 kHz, which was superimposed with a certain DC bias, and the computer both displayed the output sound pressure of 1 kHz and 2 kHz.

**Figure 8:**
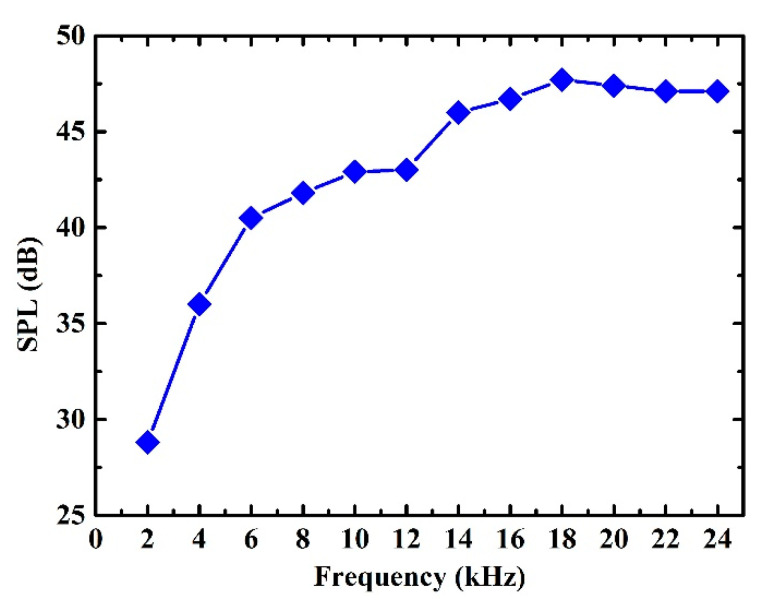
Relationship of SPL with the frequency.

**Figure 9:**
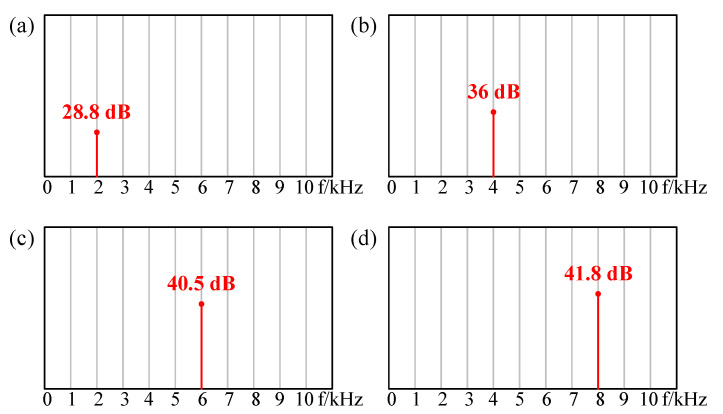
The SPL and the frequency of sound pressure under AC excitation with frequency at (**a**) 1 kHz, (**b**) 2 kHz, (**c**) 3 kHz and (**d**) 4 kHz.

For pure AC excitation, as shown in Figure 8, the relationship of the sound pressure level with the frequency of the graphene speaker was measured. The measured results show that the SPL in low-frequency increases rapidly with the frequency, and the SPL in high-frequency increases slowly with the frequency. The measured results are in good agreement with the theoretical prediction [27]. In order to study the frequency doubling effect, the measurement was carried out under with AC excitation of 10 V. As shown in Figure 9a, when the input AC frequency was 1 kHz, the output sound pressure frequency was 2 kHz and the SPL was 28.8 dB, which means that the frequency of the generated sound was twice that of the input AC excitation. When the input AC frequency was 2 kHz, 3 kHz, and 4 kHz, respectively, Figure 9b–d also verify the frequency doubling effect, which is consistent with the Equation (2).

### 4.1. DC Smaller Than AC

**Figure 10:**
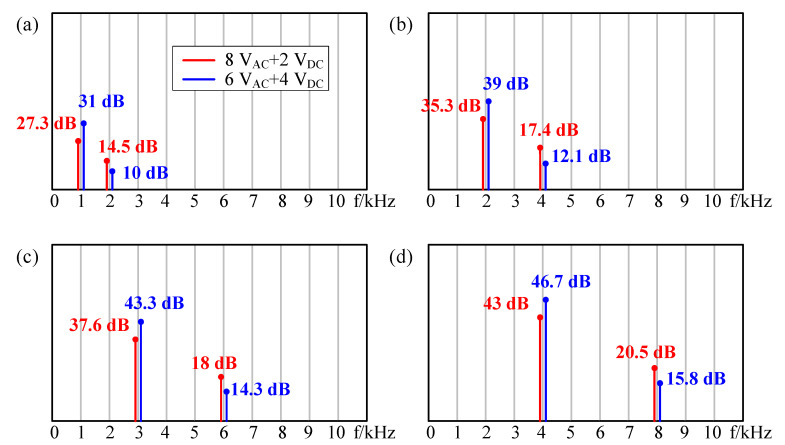
The results after DC excitation were smaller than those with AC excitation at frequencies of (**a**) 1 kHz, (**b**) 2 kHz, (**c**) 3 kHz, and (**d**) 4 kHz.

Firstly, a large AC signal and a small DC bias were used as excitation. As shown in Figure 10, the red lines indicate that the input excitation signal is 8V_AC_ + 2V_DC_, and the blue lines indicate that the input excitation signal is 6V_AC_ + 4V_DC_. As shown in Figure 10a, when the input AC frequency was 1 kHz, the frequency of sound pressure was both 1 kHz (single frequency component) and 2 kHz (double frequency component). However, the double frequency component was smaller than the single one (red one: 27.3 dB @ 1 kHz and 14.5 dB @ 2 kHz; blue one: 31 dB @ 1 kHz and 10 dB @ 2 kHz), that is, the single frequency was dominant and the double frequency was suppressed. With the increasing of DC bias, the frequency doubling effect was suppressed better. This is consistent with the results of Figure 3. When the input AC frequency was 2 kHz, 3 kHz, and 4 kHz, respectively, the same conclusion could be obtained according to Figure 10b–d.

### 4.2. DC Equal to AC

**Figure 11:**
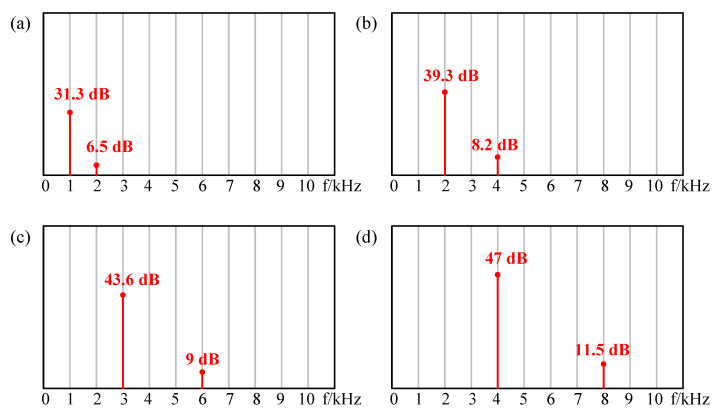
The results excited by DC equal to AC with frequency at (**a**) 1 kHz, (**b**) 2 kHz, (**c**) 3 kHz, (**d**) 4 kHz.

Then, the same amplitude of DC bias excitation with AC excitation (5V_DC_ + 5V_AC_) was applied as the excitation, as shown in Figure 11. The single frequency component and double frequency component of sound pressure exist simultaneously. As shown in Figure 11a, the SPL was 31.3 dB @ 1 kHz and 6.5 dB @ 2 kHz. Compared with Figure 9a, the double frequency component decreased, which indicates that it had better suppression effect than the case of DC being less than AC, which is consistent with the results of Figure 4. The same conclusion can be obtained according to Figure 11b–d.

### 4.3. DC Larger Than AC

**Figure 12:**
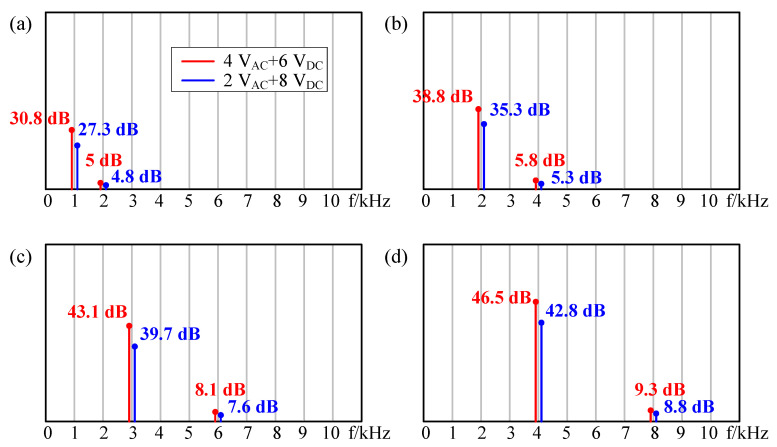
The results with DC excitation were larger than those AC excitation at frequencies of (**a**) 1 kHz, (**b**) 2 kHz, (**c**) 3 kHz, and (**d**) 4 kHz.

Finally, a small AC signal and a large DC bias were used as excitation. As shown in Figure 12, the red lines indicate that the input electric signal was 4V_AC_ + 6V_DC_, and the blue lines indicate that the input excitation signal was 2V_AC_ + 8V_DC_. As shown in Figure 12a, the single frequency component and the double frequency component of the sound pressure still existed at the same time (red one: 30.8 dB @ 1 kHz and 5 dB @ 2 kHz; blue one: 27.3 dB @ 1 kHz and 4.8 dB @ 2 kHz), but the double frequency component almost approached to 0, which can be neglected. With the increasing of DC excitation, the change of the suppression on double frequency was no longer obvious, which is consistent with Figure 5. The same conclusion can be obtained according to Figure 12b–d. Therefore, the frequency doubling effect can be suppressed by superimposing a larger DC bias excitation on the AC excitation.

## 5. Conclusions

In summary, the frequency doubling effect of thermoacoustic speakers was studied, and a method to suppress the frequency doubling effect was analyzed in this work. When the frequency of the input signal increases, the frequency doubling effect affects the performance of the thermoacoustic speakers. Three different relative amplitudes of DC excitation and AC excitation were researched: (1) when DC is less than AC; (2) when DC is equal to AC; (3) when DC is greater than AC. The measurement results show that the double frequency component was about 10dB when excited by 5V_AC_ and 5V_DC_, and less than 10 dB when excited by 4V_AC_ and 6V_DC_. This proves that the frequency doubling effect can be well suppressed by superimposing a larger DC excitation on the AC excitation. Therefore, this work has certain reference value for the theoretical research on the thermoacoustic effect and the application of thermoacoustic speakers in more fields.

## Data Availability

The data that support the findings of this study are available within the article.
